# The role of self-care interventions on men’s health-seeking behaviours to advance their sexual and reproductive health and rights

**DOI:** 10.1186/s12961-020-00655-0

**Published:** 2021-02-17

**Authors:** Manjulaa Narasimhan, Carmen H. Logie, Kevin Moody, Jonathan Hopkins, Oswaldo Montoya, Anita Hardon

**Affiliations:** 1 Department of Sexual and Reproductive Health and Research, UNDP/UNFPA/UNICEF/WHO/World Bank Special Programme, World Health Organization, 20 Avenue Appia, 1211 Geneva 27, Switzerland; 2 Factor-Inwentash Faculty of Social Work, University of Toronto, 246 Bloor Street W, Toronto, ON M5S 1V4 Canada; 3 Consultant, Amsterdam, The Netherlands; 4 U-Turn, Cape Town, South Africa; 5 MenEngage Alliance Global Secretariat, 1875 Connecticut Avenue. Floor 10, Washington, D.C. 20009 United States of America; 6 Institute for Advanced Studies and Anthropology Department, University of Amsterdam, Nieuwe Achtergracht 166, 1018 WV Amsterdam, The Netherlands

**Keywords:** Sexual and reproductive health, Men, Self care, Health behaviours, Health practices

## Abstract

**Background:**

Self-care interventions are influencing people’s access to, expectation and understanding of healthcare beyond formal health delivery systems. In doing so, self-care interventions could potentially improve health-seeking behaviours. While many men proactively engage in maintaining and promoting their health, the focus on men’s health comes from the recognition, at least partially, that male socialization and social norms can induce men and boys to have a lower engagement in institutionalized public health entities and systems around their sexual and reproductive health and rights, that could impact negatively on themselves, their partners and children.

**Main text:**

A research agenda could consider the ways that public health messaging and information on self care practices for sexual and reproductive health and rights could be tailored to reflect men’s lived realities and experiences. Three examples of evidence-based self-care interventions related to sexual and reproductive health and rights that men can, and many do, engage in are briefly discussed: condom use, HIV self-testing and use of telemedicine and digital platforms for sexual health. We apply four core elements that contribute to health, including men’s health (people-centred approaches, quality health systems, a safe and supportive enabling environment, and behaviour-change communication) to each intervention where further research can inform normative guidance.

**Conclusion:**

Engaging men and boys and facilitating their participation in self care can be an important policy intervention to advance global sexual and reproductive health and rights goals. The longstanding model of men neglecting or even sabotaging their wellbeing needs to be replaced by healthier lifestyles, which requires understanding how factors related to social support, social norms, power, academic performance or employability conditions, among others, influence men’s engagement with health services and with their own self care practices.

## Introduction

Rapid advances in the development and access to health information, products and interventions which can be used by individuals outside of a formal health setting are increasing opportunities for improved choice and informed sexual and reproductive health decision-making. These self-care interventions are often popular because they are aligned with people’s daily lives and provide the privacy and autonomy people desire, and could reduce moral judgements, stigma and discrimination within health facilities. For example, in settings where certain sexual practices are stigmatized and criminalized, such as sex work or same-sex sexual practices, HIV self-testing can offer an important, safer and more confidential additional option for knowing one’s status. However, some self-care interventions can cause harm, including for instance, products that are not evidence-based but available within the vibrant market for sexual enhancement and virility products. In acknowledgement of the ever-increasing availability of self-care interventions, including through pharmacies, and digital health platforms, the WHO global *Consolidated guidelines on self-care interventions* highlights people-centred approaches to advance health and well-being in the context of evidence-based, quality interventions and a safe and supportive enabling environment [1].

Self-care interventions are influencing people’s access to, expectation and understanding of healthcare beyond formal health delivery systems. In doing so, they could potentially improve health-seeking behaviours. This article aims to outline considerations for a research agenda to advance men’s sexual and reproductive health and rights using evidence-based examples of self-care interventions.

## Why do we need increased research focussed on men’s self-care interventions for sexual and reproductive health?

Despite sociocultural pressures to neglect their health, many men and boys proactively engage in maintaining and promoting their health and may often address their sexual and reproductive health and rights from different perspectives to women and girls. Beyond the attitude of men’s dominant position of power and their attitude around controlling women's health, there is greater room for positivity and consideration of the different approaches for men and boys to engage in better health practices. Men and boys may not have a lower engagement in health in general, but rather perhaps they have a lower engagement in institutionalized public health entities and systems that are due to a range of issues including health systems and social norms.

Access and uptake of self-care interventions is not only important to improve men’s own health, but evidence shows that engaging men in their own health care can also lead to better health outcomes in their partners and children, with men who are more involved as fathers and caregivers more likely to have better health [2, 3]. Throughout this article we use the term men applying an intersectional perspective, where we consider sexual diversity (gay, bisexual, and other men who have sex with men (MSM)) and gender diversity (transgender, gender non-conforming, and non-binary persons), and their interplay with other social categories such as race, ethnicity, class, sex work involvement, and immigration status. Evidence also suggests that meeting the sexual and reproductive health needs of men results in lower rates of sexually transmitted infections in men and women, in lower rates of unintended pregnancy, and in healthier and more satisfying personal and family relationships [4]. The man plays a significant—up to 50%—role in heterosexual couples experiencing infertility/sub-fertility and consequently are engaged in diverse ways when approaching the health system to attempt to become a father [5]. Multiple issues signal the importance of placing men’s sexual and reproductive health centre stage in the political and research agenda, including the need to improve transgender men’s experiences in seeking gynecological and reproductive care [6]; the importance of addressing reproductive dysfunction induced from excessive exercise [7]; and, the consideration of men’s other health issues, including high body mass index, lifestyle, and environmental factors that impact on reproductive health parameters [8].

The focus on men’s health also comes from the recognition, at least partially, that men’s socialization and social norms contribute to lower engagement in health promoting practices (such as regular medical appointments) that could impact negatively on themselves, their partners and children [9]. It has also been driven by a growing body of evidence that provides a better understanding of how gender intersects with other social, economic, environmental, political and cultural determinants influencing exposure to risk factors and interactions with health systems [10].

## What are core components of a self-care research agenda?

The WHO conceptual framework on self-care interventions, expert WHO consultations, and outcomes of a global values and preferences survey and focus group discussions with vulnerable and marginalized communities contributed to the development of WHO’s normative guidance on self-care interventions [11, 12]. The elaboration of this guideline also highlighted core aspects of accessing or using self-care interventions that could positively influence health-seeking behaviours. Elaboration of men’s health seeking behaviour, in particular, was recommended as an area for further research by the Scientific and Technical Advisory Group of the Special Programme of Research, Development and Research Training in Human Reproduction. Four aspects as it may relate to health include: people-centred approaches, quality health systems, a safe and supportive enabling environment, and behaviour-change communication.

- People-centred approaches: Meaningful engagement of men and boys to ensure access to opportunities, programmes and services can potentially advance their health, including their sexual and reproductive health and rights. This engagement is relevant from early in life and throughout their life course.
- Quality health systems: Self-care interventions cannot be implemented without a strong link with health systems and even in settings where the health system may be weak, this link remains important.
- Promoting a safe and supportive enabling environment: Uptake of self-care interventions should not be driven by lack of other options for individuals who are either marginalized, criminalized or otherwise made vulnerable because of their circumstances and/or identities. Implementing comprehensive and integrated sexual and reproductive health and rights to meet the health needs and rights of men and boys requires overcoming barriers at the individual, interpersonal, community and societal levels to service uptake and use and continued engagement.
- Promoting behaviour-change communication: The ability of people to achieve sexual and reproductive health and rights and wellbeing depends, among other things, on their access to comprehensive information about sexuality and reproduction, their knowledge about the risks they face, and their vulnerability to the adverse consequences of unsafe sexual activity.

## Proposing research considerations for self care in men’s sexual and reproductive health

In general, women are more prone to engage in healthy practices than men—including self care [13]. In Europe, compared to women, and across socio-economic groups, men go less frequently to the doctor’s, consistently report less unmet health-care needs, and are more likely to smoke, demonstrate less healthy dietary patterns, as well as higher alcohol consumption levels, rates of injuries and interpersonal violence perpetration [14]. Globally, life expectancy at birth in 2016 was 74.2 years for females and 69.8 years for males, and the pattern of higher risk of mortality for men compared to women is consistent across regions and countries [15].

A research agenda could consider the ways that public health messaging and information on self care practices for sexual and reproductive health could be tailored to reflect men’s lived realities and experiences. For instance, factors thought to influence men’s health-seeking behaviour include traits associated with masculinity, including suppression of emotion (stoicism), independence, self-reliance and dominance [16]. Belief systems around masculinity and the degree to which individuals subscribe to these beliefs can differ depending on cultural context and can vary among groups of men and throughout their life-course. The conflict between these ideologies that might idealize characteristics of strength and dominance could affect healthy courses of action. For instance, online pornographic images could result in increased sexual violence [17]. Risk-taking factors in men also contributes to more injury-related morbidities and decreased likelihood of accessing health services [18].

Health system challenges include a lack of clear entry points for men that women generally have to access sexual and reproductive health services—for instance, men do not interface with healthcare providers at the same frequency as their female counterparts who are far more likely to access contraceptive services and may access pregnancy services. Poor or reduced access and other barriers to health care can act as deterrents. This includes for instance, lack of extended opening hours and facilities-based health care for men who work outside their communities during the day [19–21]. In some settings, recommended health services and estimated visits required across the reproductive lifespan (between 15 and 44 years) show that women attend the health facility between 176 and 433 times and men to attend just 30 times [22, 23]. There are also challenges regarding respectful communication and competence of health workers to attend to transgender men, and stigma, discrimination, and criminalization of sexual and gender minority men could also result in avoidance of formal healthcare settings [24, 25]. The combination of social, behavioural and physical factors, and health systems challenges provides a better understanding of barriers men face in accessing health services for their sexual and reproductive health needs [18, 26, 27]. However, when there are services dedicated to men, studies have shown that there is an increase in health seeking behaviours [28], that underscores the great potential of self-care strategies in increasing safe and private sexual and reproductive health and rights options for men.

## Opportunities for improved health outcomes for men through self-care interventions for sexual and reproductive health

Three examples of evidence-based self-care interventions related to sexual and reproductive health and rights that men can, and many do, engage in are briefly discussed.

### Condom use

Condoms remain the most common male contraception and one of the few ways in which men take an active role in contraception and prevention of sexually transmitted infections. Despite generally increasing trends in condom use over the past two decades, substantial variations and gaps remain. Despite concerns of inconsistent or incorrect use, and rejection of this method, its acceptance is gradually growing, especially among young men [29]. Further funding and programmatic support are needed to promote access and use of condoms as well as supportive policies.

### HIV self-testing

In 2018, 79% of people living with HIV knew their status, and availability of HIV self-testing increased opportunities for knowing sero-status among men. Given the increased privacy and confidentiality it offers, HIV self-testing provides an important health service entry point [30]. A programme in Kenya also showed cost-effective increases in men’s HIV testing when women attending prenatal visits were given HIV self-test kits to give to their partners. This study showed women’s willingness to distribute the HIV self-tests was high, as was the uptake of HIV self-testing by men [31]. Yet this may be a context specific outcome that also involves concerns about increasing the burden of prevention onto women, along with the risk of intimate partner violence. There is evidence on the benefits of self-testing for HIV among gay men and other MSM [32]. It signals the need to explore experiences and preferences for partner HIV self-testing among MSM across global regions.

### Telemedicine and digital platforms for sexual health

The internet may be among the first places that men go to seek information, and for many young people, digital technology and mobile applications are an integral part of their lives [33]. Adapting public health programmes to be designed and delivered in a way that is flexible and iterative is increasingly important [34]. For instance, young people from ethnic minority groups found acceptability in online information regarding perceptions of smartphone-enabled self-testing and online care for sexually transmitted infections with the three main factors for choosing a service including speed, convenience and privacy, the latter making online solutions attractive for men who were more likely to use the internet for sexual partners [35, 36].

We apply the four core elements (people-centred approaches, quality health systems, a safe and supportive enabling environment, and behaviour-change communication) to each intervention where further research can inform normative guidance (Table1).

**Table 1:** Considerations for research and programming for selected self-care interventions to support men’s sexual and reproductive health and rights

Type of self-care intervention	Considerations for a self care agenda for men’s sexual and reproductive health and rights			
	People centred	Quality health system	Safe and supportive environment	Behaviour change communication
Condom use	Comprehensive sexuality education for all young people can build the foundation of safer sex practices (including condom use) and healthy relationships (including safer sex communication) to highlight that condoms protect for HIV alongside other sexually transmitted infections, and pregnancy^a^ Increased access to condoms and water- and silicone-based lubricants through diverse outlets, including outside of traditional health facilities.^b^	Access and promotion of condoms for diverse men within health care settings and through community health workers Condom availability at sexual and reproductive health clinics to link to sexually transmitted infections, including HIV tests, treatment and prevention services Extension of health services to provide condoms in non-traditional settings where men congregate	Supportive laws and policies for diverse men (and women) for the possession of condoms and lubricants Reduced stigma and discrimination experienced by MSM, transgender men, along with reducing other intersecting forms of stigma, such as sex work stigma and racism, as well as within people with a migration background Actively working towards a human-rights based approach to eliminate policies which criminalize practices such as carrying condoms	Social marketing tailored to reflect the priorities, preferences and experiences for diverse men (including sexually, gender, and racially diverse) to support condom uptake
HIV self-testing	Campaigns can highlight the benefits of autonomy, build self-efficacy for testing, and increase availability for self-tests (e.g. free or very low cost) to lower access thresholds for testing Increased awareness, knowledge and access to HIV self-testing options for men and boys from vulnerable populations, such as sex working men	Established links to confirmatory testing; treatment, if needed; and, services to support prevention strategies, including pre-exposure proplylaxis for HIV-negative men Knowledge-sharing and information campaigns, including brief sexuality counselling and communication programmes [37]	Increased number of health workers trained and supportive agencies for men who are diverse men Reduced stigma and discrimination experienced by diverse vulnerable and marginalized men, including sex workers and men-who-have-sex-with-men Understand and address men’s barriers/facilitators to self-testing, and preferred delivery methods and points of access	Targeted outreach for key populations and higher-risk populations with brief sexuality communication HIV self-testing kits provided to women at health facilities to encourage men to test at home with a view to monitor and prevent potential undesired increases on the burden on women, including potential increase in violence
Telemedicine and digital platforms for sexual health	Telemedicine to link clients and providers with tailored information and services where and when they need it	Data security, particularly for vulnerable and marginalized men, and strengthened linkages among medical laboratories, facilities, practitioners and pharmacies	Delivery of sexual and reproductive health services through digital health technologies, for increased accessibility, safety and privacy Reduced intersectional stigma and discrimination experienced by men	Can use motivational prompts, behavioural science Link to common uses of technology by men, including online dating services Can address how men are affected by images and messages of dominant masculinity norms

## Conclusion

Improving the health and wellbeing of smen and that of women are complementary objectives that require a systematic approach to gender equality. Engaging men and facilitating their participation in self care can be an important policy intervention to achieve global sexual and reproductive health and rights goals.

In the context of sexual and reproductive health services, men often lack the clear entry points that women can access due to the design and focus of health care systems. Self-care interventions could provide opportunities to improve men’s health seeking behaviours and thereby improve their health outcomes. Self-care interventions can contribute to increased options, agency and autonomy in health decision-making and have the potential to reinforce positive health seeking behaviours, including among transgender men and men-who-have-sex-with-men. Offering self-care interventions through community-led mechanisms is relevant and can increase outreach to vulnerable and marginalized and underserved populations. Implementing self-care interventions within a safe and supportive enabling environment could provide additional access points for men by positively addressing their health rights, needs and priorities.

Limited transferability can be claimed given that we have only focussed on three examples of self-care interventions. Despite this limitation, the findings are insightful and of practical importance for programmes aimed at advancing men’s health outcomes. Further research on bridging the gap between individual, communities and the formal health sector on self-care interventions for men is needed.

Men have an important role to play in the care of their own health and that of their partners and families, as well as in the gender equality agenda. The longstanding model of men’s neglecting or even sabotaging their wellbeing needs to be replaced by healthier lifestyles, which requires understanding how factors related to social support, social norms, power, academic performance or employability conditions, among others, influence men’s engagement with health services and with their own self care practices. This review highlights how public health can appropriately engage with men and boys, with regards to their sexual and reproductive health and rights. Further research in this area, and providing better access to evidence-based, self-care interventions can support the right to health for all.
